# Transcriptional regulation of Glis2 in hepatic fibrosis

**DOI:** 10.1038/s12276-023-01031-y

**Published:** 2023-07-03

**Authors:** Huan-Yu Gong, Peng-Cheng Zhou, Hao-Ye Zhang, Li-Min Chen, Yang-Mei Zhou, Zhen-Guo Liu

**Affiliations:** 1 Department of Infectious Disease, the Third Xiangya Hospital, Central South University, Changsha, 410013 Hunan PR China; 2 Hunan Key Laboratory of Viral Hepatitis, Xiangya Hospital, Central South University, Changsha, 410008 Hunan PR China

**Keywords:** Cell biology, Cancer

## Abstract

The role of Gli-similar 2 (Glis2) in hepatic fibrosis (HF) is controversial. In this study, we focused on the functional and molecular mechanisms involved in the Glis2-mediated activation of hepatic stellate cells (HSCs)—a milestone event leading to HF. The expression levels of Glis2 mRNA and protein were significantly decreased in the liver tissues of patients with severe HF and in mouse fibrotic liver tissues as well as HSCs activated by TGFβ1. Functional studies indicated that upregulated Glis2 significantly inhibited HSC activation and alleviated BDL-induced HF in mice. Downregulation of Glis2 was found to correlate significantly with DNA methylation of the Glis2 promoter mediated by methyltransferase 1 (DNMT1), which restricted the binding of hepatic nuclear factor 1-α (HNF1-α), a liver-specific transcription factor, to Glis2 promoters. In addition, the enrichment of DNMT1 in the Glis2 promoter region was mediated by metastasis-associated lung adenocarcinoma transcriptor-1 (MALAT1) lncRNA, leading to transcriptional silencing of Glis2 and activation of HSCs. In conclusion, our findings reveal that the upregulation of Glis2 can maintain the resting state of HSCs. The decreased expression of Glis2 under pathological conditions may lead to the occurrence and development of HF with the expression silencing of DNA methylation mediated by MALAT1 and DNMT1.

## Liver disease: Regulatory protein helps prevent fibrosis

A regulatory protein that helps keep specialized liver cells from becoming activated offers a potential new therapeutic target for treating the thickening or scarring of tissue known as fibrosis. Working with human biopsy samples and mouse models, Zhen-Guo Liu and colleagues from Central South University in Changsha, China, found that levels of a protein called GLIS2 are significantly reduced in fibrotic liver tissues compared to healthy tissues. In fibrotic tissues, the gene encoding GLIS2 is tagged with methyl groups that prevent the binding of another regulatory protein needed for gene expression. GLIS2 levels then drop and specialized liver cells known as hepatic stellate cells undergo a transformation that fuels the build up of fibrosis. Drugs that boost activity of GLIS2 could help maintain these liver cells in a less active state and thus prevent fibrosis.

## Introduction

Hepatic fibrosis (HF) is a wound healing process that leads to excessive extracellular matrix (ECM) accumulation and dysregulation of liver function^1^. HF has been recognized as a pathological response to almost all types of chronic liver disease, such as viral hepatitis, fatty liver disease, and metabolic and autoimmune disorders of the liver^2^. Even if HF is partially reversible, it may progress to end-stage cirrhosis or even hepatocellular carcinoma^3^. Activation of hepatic stellate cells (HSCs) is known to play a key role in the biological processes of HF. After liver injury, quiescent HSCs transdifferentiate into myofibroblast-like cells, which have an increased ability to proliferate, migrate, and express fibrosis markers^4^. Therefore, elucidating the mechanisms of fibrotic occurrence and development in which HSCs participate is important for the treatment of this disease.

The Krüppel-like zinc finger protein Gli-similar 2 (Glis2) is a member of the Glis family, which contains three transcription factors: Glis1, Glis2, and Glis3^5^. Glis proteins have been implicated in several pathologies, including cystic kidney disease, diabetes, hypothyroidism, fibrosis, osteoporosis, psoriasis, and cancer^6–8^. Among the Glis family proteins, Glis2 has more often been implicated in fibrotic diseases. For example, in renal fibrosis, high expression of Glis2 is correlated with the inhibition of epithelial–mesenchymal transformation (EMT), mediated by inhibition of the zinc finger protein Snail, which maintains the epithelioid phenotype of renal cells^9,10^. However, a study of nonalcoholic steatohepatitis induced by a high-fat and high-sugar diet in mice reached the opposite conclusion, finding that Glis2 knockout delayed the occurrence of HF in mice under this induction condition^11^. Overall, although there are various opinions on the role of Glis2 in fibrosis, it is undeniable that Glis2 is closely related to fibrosis.

New evidence suggests that lncRNA-mediated DNA methylation affects HF^12–14^. Coincidentally, we found a CpG island ~2000 bp in length in the promoter region of Glis2, in which methylation regulation of Glis2 promoters may occur, thereby affecting its transcription. There are eight lncRNAs closely related to hepatic diseases according to the Lnc2Meth database (http://bio-bigdata.hrbmu.edu.cn/Lnc2Meth/cu_transcript.jsp), among which the lncRNA metastasis-associated lung adenocarcinoma transcript 1 (MALAT1) was reported to be highly expressed in fibrotic liver tissues compared with normal liver tissues^15–17^. MALAT1 appears to be an important epigenetic regulator of DNA methylation. For instance, it regulates mitochondrial metabolism of hepatocellular cancer cells by altering CpG methylation patterns of mtDNA16^18^, or it impedes proliferation and inflammation in fibroblast-like synoviocytes through CTNNB1 promoter methylation in rheumatoid arthritis^19^. However, the role of MALAT1-mediated DNA methylation in HF remains unclear.

In this study, we demonstrated that Glis2 is downregulated in fibrotic liver and activated HSCs, which can be attributed to MATAL1-mediated DNA methylation modification in the promoter region of Glis2. As a result, the transcriptional activator of Glis2, hepatocyte nuclear factor 1-α (HNF1-α), fails to bind to the hypermethylated promoter, leading to a decline in Glis2 transcription. These findings enrich our understanding of the roles of Glis2 in HF and provide promising targets for intervention.

## Materials and methods

### Clinical specimen collection

Human fibrotic liver tissues (*n* = 6) were obtained from patients who underwent liver biopsy at the Third Xiangya Hospital of Central South University, Changsha, Hunan, China. The inclusion criteria were chronic hepatitis B patients with varying degrees of liver fibrosis, excluding patients with chronic liver disease caused by alcoholic liver disease (ALD), nonalcoholic fatty liver disease (NAFLD), autoimmune hepatitis (AIH), drug-induced liver disease (DILD) and other causes. All patients signed informed consent, and this study was approved by the IRB of the Third Xiangya Hospital, Central South University (2020-S076). The diagnostic data of the patients are shown in Table 1.

**Table 1:** Diagnostic data of the included patients.

NO.	Age	Gender	METAVIR	Platelets(×10 ^9^ /L)	ALT(U/L)	AST(U/L)	TB(μmol/L)	Albumin (g/L)	LSM(kPa)	Fibrosis- 4 index
1	51	Female	F3 (Moderate)	136.5	56.4	34.1	14.6	41.5	14.6	1.7
2	43	Female	F2 (Moderate)	186.7	36.6	42.4	11.4	42.6	10.9	1.6
3	56	Male	F3 (Moderate)	102.4	68.8	42.9	20.6	38.9	17.4	2.8
4	42	Male	F4 (Severe)	94.7	96.3	81.6	43.4	36.3	19.6	3.7
5	37	Male	F3 (Moderate)	113.8	63.6	46.8	23.6	41.6	16.9	1.9
6	58	Male	F4 (Severe)	86.5	107.5	82.6	53.7	35.8	23.4	5.3
mean	47	/	/	120.1	71.5	55.1	27.9	39.4	17.1	2.8
SD	8	/	/	37.0	26.1	21.3	16.9	2.9	4.3	1.5

### Cell culture and treatment

JS-1 cells (mouse hepatic stellate cell line), purchased from Fenghui Biotechnology (Changsha, China, CL0417), were cultured in DMEM (Gibco, 11965118) with 10% fetal bovine serum (FBS; ExCell Bio, FSP500), 100 U/mL penicillin, and 100 μg/mL streptomycin (Gibco, 15140163) at 37 °C and 5% CO_2_ in humidified air. Cells were serum-starved with 0.5% FBS for 12 h prior to TGFβ1 treatment (5 ng/mL, Peprotech, 100-21)^20^. The isolation of primary hepatic astrocytes was performed as described^21^.

### Bile duct ligation (BDL) liver injury mouse model

C57BL/6 mice (*n* = 5 in each group; 20 ± 2 g b.w.) were purchased from Hunan SJA Laboratory Animal Co. Ltd. (Hunan, China). HF was induced with BDL^22^. Briefly, mice in each group were fasted for 1 d before surgery and then anesthetized with 10% chloral hydrate intraperitoneal injection at 0.04 mL/kg. The abdominal fur was shaved, and the anesthetized mice were subjected to midline laparotomy. The common bile duct was exposed and ligated at two points, and the surgical incision was closed. Mice were kept at 37 °C on a heated hot plate until they were fully awake. For the sham group, the bile duct of mice was manipulated without ligation. Mice were sacrificed with cervical dislocation, and the liver tissues were harvested and subjected to histological analysis. The liver function indicators of each mouse are shown in Table 2.

**Table 2:** Data of BDL-induced mice liver function index.

Groups	Sham							BDL						
Index	1	2	3	4	5	mean	SD	1	2	3	4	5	mean	SD
AST (U/L)	64.7	42.8	48.4	58.1	39.2	50.6	10.6	77.4	91.2	57.9	76.5	67.1	74.0	12.5
ALT (U/L)	41.2	40.8	40.2	41.8	38.9	40.6	1.1	192.5	188.4	198.4	183.1	184.7	189.4	6.2
TB (μmol/L)	3.1	2.4	1.7	1.2	8.3	3.3	2.9	120.5	117.9	112.3	129.2	122.1	120.4	6.2
Hydroxyproline (μg/g)	60.1	61.0	50.7	48.3	40.4	52.1	8.6	144.3	155.6	198.9	211.3	157.4	173.5	29.6

### CCl4 liver injury mouse model and animal care

C57BL/6 mice (*n* = 5 in each group; 20 ± 2 g b.w.) were used to construct liver fibrosis models by intraperitoneal injections of carbon tetrachloride (CCl_4_; 0.5 µL/g, dissolved in corn oil at a ratio of 1:3), and only corn oil was injected as a control. Mice were sacrificed with cervical dislocation, and the liver tissues were harvested and subjected to histological analysis. The liver function indicators of each mouse are shown in Table 3.

**Table 3:** Data of CCL4-induced mice liver function index.

Groups	Sham							CCL4						
Index	1	2	3	4	5	mean	SD	1	2	3	4	5	mean	SD
AST (U/L)	28.8	42.2	58.4	44.3	39.7	42.7	10.6	74.3	66.3	80.2	81.3	85.1	77.4	7.3
ALT (U/L)	48.9	37.8	52.1	59.0	45.5	48.7	7.9	138.8	147.7	136.3	118.6	122.5	132.8	12.0
TB (μmol/L)	20.9	10.7	9.5	19.3	19.7	16.0	5.5	32.5	38.9	47.8	55.4	60.3	47.0	11.4
Hydroxyproline (μg/g)	69.3	40.2	51.3	58.9	30.2	50.0	15.3	139.7	173.4	197.4	201.3	164.5	175.3	25.3

Animal experiments in this study were conducted according to the National Institutes of Health Guide for the Care and Use of Laboratory Animals and were approved by the IRB of the Third Xiangya Hospital, Central South University (2020-S076).

### Isolation of primary hepatocytes and HSCs

Primary mouse HSCs were isolated by pronase/collagenase perfusion digestion, followed by density gradient centrifugation^23^ Briefly, liver tissues were initially digested in situ with 0.05% pronase E (Roche, Shanghai, China) and 0.03% collagenase type IV (Sigma–Aldrich, Shanghai, China) and then further digested with collagenase type IV, pronase E, and DNase I (Roche) at 37 °C in a shaking bath for 20 min. HSCs were isolated from nonparenchymal cells using Nycodenz solution (Sigma–Aldrich) at 4 °C due to the presence of massive amounts of vitamin A-storing lipid droplets. Primary HSCs were cultured in high-glucose Dulbecco’s modified Eagle’s medium containing 10% FBS and 1% penicillin–streptomycin and were maintained in a humidified incubator with 5% CO_2_ at 37 °C.

The isolation and culture of primary hepatocytes was performed according to the technical patent of this research team (2017.03.08, National Invention Patent, CN201510418641.3).

### Histochemical staining

Liver tissues were fixed in 4% paraformaldehyde (Servicebio, G1101-500ML), embedded in paraffin, and sectioned at 5 μm. Paraffin-embedded liver sections were dewaxed, rehydrated, and stained with H&E, Masson’s stain, and Sirius Red, as described^24^.

### Immunohistochemistry

Paraffin-embedded liver sections were dewaxed, rehydrated, and subjected to antigen retrieval. The sections were blocked with 10% normal goat serum and incubated with primary antibody at 4 °C overnight. The signal was visualized using the mouse- and rabbit-specific HRP/AEC IHC detection kit (Servicebio, Wuhan). The primary antibodies used in immunohistochemistry (IHC) are listed in Table 4.

**Table 4:** Antibodies used in this study.

Antibody	Vendor	Catalog no.	Working dilution
Glis2	Invitrogen	PA5-72849	1:1000 (WB); 1:100 (IF); 1:100 (IHC)
α-SMA	Abcam	ab7817	1:2000 (WB); 1:100 (IF); 1:100 (IHC)
Desmin	Abcam	ab15200	1:1000 (WB); 1:100 (IF); 1:100 (IHC)
CoL1A1	Abcam	ab34710	1:1000 (WB); 1:100 (IF)
p75NTR	Abcam	ab52987	1:1000 (WB); 1:50 (IF)
DNMT1	Abcam	ab13537	1:50 (ChIP); 1:50 (RIP)
DNMT3b	CST	57868 S	1:50 (ChIP)
DNMT3a	CST	49768 S	1:50 (ChIP)
HNF1α	Abcam	ab181604	1:500 (WB); 1:50 (ChIP)
β-actin	Abcam	ab8226	1:2000 (WB)
GAPDH	CST	92310	1:2000 (WB)

### Hepatic fibrosis evaluation

Hydroxyproline in liver tissues and aspartate aminotransferase (AST), alanine aminotransferase (ALT), and TB in serum were evaluated using commercial kits (Abcam, Cambridge, UK). Serum levels of IL-1β, IL-8, TNF-α, and CCL-2 were evaluated using commercial ELISA kits (R&D Systems, Minneapolis, MN, USA) according to the manufacturer’s protocols.

### Cell Counting Kit-8 (CCK-8) assay

Cell proliferation was detected using the CCK-8 assay (Beyotime Biotechnology, C0037). Briefly, HSCs were plated in 96-well plates. At 0, 24, 48, and 72 h, the CCK-8 solution was incubated with cells at 37 °C for 2 h. Absorbance was measured at 450 nm using a microplate reader (Bio-Rad Laboratories, Hercules, CA, USA).

### EdU incorporation assay

The EdU incorporation assay was conducted using the BeyoClick EdU-488 cell proliferation kit (Beyotime Biotechnology, C0071S). Briefly, HSCs were incubated with EdU for 12 h. Cells were fixed with 4% PFA for 15 min and incubated first with 0.3% Triton X-100 for 15 min and then with Click Additive Solution. Nuclei were visualized by DAPI. Images were photographed using a confocal laser scanning microscope (Carl Zeiss, Jena, Germany).

### Transwell migration assay

Cell migration was monitored using the Transwell migration assay. Briefly, HSCs were seeded in the upper Transwell chambers (Corning, Inc., Corning, NY, USA) and cultured in serum-free DMEM. The lower chambers were filled with DMEM containing 10% FBS. After 48 h, the migrated cells were fixed with 4% PFA and stained with crystal violet. Stained cells in three random fields of each chamber were counted under a microscope (Carl Zeiss, Jena, Germany).

### qRT‒PCR

Total RNA was isolated from tissues and cells using TRIzol reagent (Invitrogen, 15596-026) and reverse transcribed using a cDNA reverse transcription kit (TaKaRa, RR037A). qRT‒PCR was performed using SYBR Green PCR Master Mix (TaKaRa, Dalian, China). GAPDH was used as an internal control for normalization. The relative expression of the target gene was determined using the 2^−ΔΔCT^ method. The primers used in qRT‒PCR are listed in Table 5.

**Table 5:** The list of prime sequence of qRT-PCR.

Pirmer	Sequence 5′-3′
hGlis2 sense	GGTGGACCATGTCAACGATT
hGlis2 anti-sense	GACGTAGGGCTTCTCACCTG
mGlis2 sense	TTCTTCTTGCCCCTGGGTTC
mGlis2 anti-sense	AGCTGGTTACACTTGGCCC
m-p75NTR sense	CAACCAGACCGTGTGTGAACCC
m-p75NTR anti-sense	CCTGGTAGTAGCCATAGGAGCATC
m-α-SMA sense	CTTCGTGACTACTGCCGAGC
m-α-SMA anti-sense	AGGTGGTTTCGTGGATGCC
mDesmin sense	GCCGACGCTGTGAACCAGGA
mDesmin anti-sense	GCGCGGCGTTCTGCTGCTCCA
hMALAT1 sense	GCTCTGTGGTGTGGGATTGA
hMALAT1 anti-sense	GTGGCAAAATGGCGGACTTT
mMALAT1 sense	GTATGTAGGCCTTTGCGGGT
mMALAT1 anti-sense	GGTTGTGCTGGCTCTACCAT
lncRNA00441 sense	CAAAGCCACTGCAACAAGAG
lncRNA00441 anti-sense	TAGGGAAGGGTTTGTGCATC
hHOTAIR sense	GCCTTTCCCTGCTACTTGTG
hHOTAIR anti-sense	AGAGCTTCCAAAGGCTAGGG
hH19 sense	TCCCGGTCACTTTTGGTTAC
hH19 anti-sense	CGATCCCCTAAACCTCCTTC
hCUDR sense	CGGGTAACTCTTACGGTGGA
hCUDR anti-sense	ATGGTGAACCCAATGGAGAG
hAS1DHRS4 sense	AGGTTGCAGTGAGCCAAGAT
hAS1DHRS4 anti-sense	GGGAACTGAAACCACTTTGC
hGIHCG sense	GTTGTGGTTGCTGGCTGTTA
hGIHCG anti-sense	GACTGGATAACCGCTTGTGA
hHULC sense	GAGTCGTCACGAGAACCAGA
hHULC anti-sense	GCCAGGAAACTTCTTGCTTG
GAPDH sense	GCTGTAGCCAAATCGTTGT
GAPDH anti-sense	CCAGGTGGTCTCCTCTGA
hGlis2 sense (for ChIP)	AGCCAGACCCATCCCTTTAT
hGlis2 anti-sense (for ChIP)	TCAGATCGCAGAGCTCAGAA
mGlis2 sense (for ChIP)	GCTTATTTCACCAGGAGGGCA
mGlis2 anti-sense (for ChIP)	AAAGCCCCAAGGACCGGGAA
hMALAT1 sense (for RIP)	GGCATTTCATCCTTCATGAAGCC
hMALAT1 anti-sense (for RIP)	TCCCATCCCTCCAAATTCCAGG
mMALAT1 sense (for RIP)	GTTCTAGTTTGAAGGTCGGCC
mMALAT1 anti-sense (for RIP)	ACGGCCGTCAACTTAACCTAC
mHNF1α sense	AGCGGGCATCCACGAAAC
mHNF1α anti-sense	TTGATCTTCATGGTGCTGGGT

### Multilabel immunofluorescence (IF)

A multicolor fluorescent labeling kit was used according to the manufacturer’s instructions (AFIHC024, AiFang Biological, Changsha, Hunan, China). In brief, coverslips were fixed in 4% PFA, permeabilized in 0.1% Triton X-100, and blocked with 1% BSA. After antigen repair, coverslips were incubated with primary antibody at 37 °C for 1 h and then with fluorescently labeled secondary antibody in the dark for another hour. Then, antigen repair and antibody incubation were repeated after washing with PBS until all antibodies to be labeled had completed an immunofluorescence operation. Finally, slips were cleaned with PBS to remove residual fluid and stained with DAPI for 10 min at 25°C. The excitation wavelengths of fluorescence of various colors used in this study are as follows: pink (ex: 670 nm), red (ex: 620 nm), green (ex: 520 nm) and blue (ex: 480 nm). Images of the specimens were captured under a KFBio slide viewer (KFBio, Ningbo, Hangzhou, China). The primary antibodies used for immunofluorescence are listed in Table 4.

### Western blotting

Protein lysates were extracted from tissues and cells using RIPA buffer (Beyotime Biotechnology, P0013B), separated by SDS‒PAGE, and transferred to PVDF membranes (Millipore, Billerica, MA, USA). The membranes were blocked with 5% nonfat milk and incubated with primary antibodies at 4 °C overnight and the corresponding HRP-conjugated secondary antibodies at room temperature for 1 h. The signals were detected by chemiluminescence using the Chemidoc system (Bio-Rad, Munich, Germany). Data were analyzed using ImageJ software, and figures were cropped. The primary antibodies used in western blotting are listed in Table 4.

### Lentiviral production and transduction

The coding sequence of Glis2 or full-length MALAT1 was cloned into pLV-CMV-MCS-EF1-ZsGreen1-T2A-Puro (Genomeditech, Shanghai, China). The empty vector served as a negative control for the overexpression experiment. The constructs were used to produce lentiviruses in 293 T cells with packaging plasmids using the HG transgene reagent (Genomeditech, Shanghai, China). Lentiviruses were harvested 72 h post-transfection and filtered through a 0.45-μm PVDF filter (Millipore). The JS-1 cells were then infected with lentiviruses for subsequent analysis. For the in vivo overexpression experiment, the recombinant adenovirus Admax-pDC316-mCMV-Glis2 (or MALAT1-shRNA)-GFP (Genomeditech, Shanghai, China) was injected through the tail vein 2 weeks after surgery. Mice were euthanized 2 weeks after virus injection, and tissue isolation and testing were performed as needed.

### Bisulfite sequencing

Bisulfite sequencing was performed to detect the methylation status of the Glis2 promoter in liver tissues and HSCs. Briefly, genomic DNA was extracted using the PureLink Genomic DNA Mini Kit (Thermo Scientific, K0512). Bisulfite conversion of genomic DNA was performed using the EpiTect Bisulfite Kit (Qiagen, 59104). The primers for bisulfite sequencing were designed using MethPrimer (Table 3). PCR products were purified and cloned into the pMD19-T vector. Four clones of each sample were sequenced.

### Chromatin immunoprecipitation (ChIP) assay

ChIP assays were performed using the SimpleChIP plus Enzymatic Chromatin IP Kit (Cell Signaling Technology, 9005 S) according to the manufacturer’s instructions. Briefly, cells were lysed after crosslinking with 1% formaldehyde. Chromatin was digested with MNase and immunoprecipitated using anti-DNMT1 (Abcam, ab13537), anti-DNMT3a (CST, 49768 S) and anti-DNMT3b (CST, 57868 S) antibodies or the corresponding normal IgG (negative control). DNA was purified and analyzed by qRT‒PCR. Positive and negative controls were included. The primers used in the ChIP–qPCR assay are listed in Table 5.

### RNA immunoprecipitation (RIP)

RIP was conducted using the Magna RIP^TM^ RNA-Binding Protein Immunoprecipitation Kit (Millipore, 17-700) according to the manufacturer’s instructions. Immunoprecipitation was performed using the anti-DNMT1 antibody (Abcam, ab13537) or normal mouse IgG (negative control). MALAT1 levels were determined by qRT‒PCR. The primers used in RIP-qPCR are listed in Table 5.

### Fluorescence in situ hybridization (FISH)/IF

The FITC-conjugated probe for MALAT1 was designed and synthesized by RiboBio (Guangzhou, China). Cells grown on coverslips were fixed with 1% formaldehyde and permeabilized with 70% ethanol at 4 °C overnight. Cells were incubated with the FISH probe and anti-DNMT1 antibody in hybridization buffer at 37 °C overnight, followed by incubation with Alexa Fluor 594-conjugated secondary antibody. Slides were mounted with Prolong Gold antifade reagent and DAPI (Invitrogen). The images were acquired using a confocal laser scanning microscope (Carl Zeiss, Carl Zeiss, Jena, Germany).

### RNA pulldown assay

RNA pulldown assays were performed using the Pierce Magnetic RNA‒Protein Pull-Down Kit (Pierce). Briefly, the 3′ end of synthesized MALAT1 was labeled with desthiobiotin using the Pierce RNA 3′ End Desthiobiotinylation Kit. Labeled MALAT1 was conjugated with streptavidin beads and incubated with protein lysates. The eluates were subjected to western blot analysis using the anti-DNMT1 antibody. Lac Z served as a negative control. For protein lysates derived from the clinical specimens and animals, the samples (*n* = 3) were pooled for analysis due to the limited sample size.

### DNA pulldown assay

A biotin-labeled double-stranded oligonucleotide probe for the Glis2 promoter sequence was synthesized. Briefly, 1 mg of nuclear protein extract was mixed and incubated with 10 μg of probe and 100 μl of streptavidin-agarose beads (Sigma, St Louis, MO). The mixtures were then centrifuged at 800×g, resuspended in 30 μl of loading buffer, and boiled at 100 °C for 5 min. The collected samples containing the bound proteins were separated by SDS‒PAGE for western blot analysis.

### Luciferase analysis

The HNF1α/GATA4/FOXA3/HNF4α expression vectors for human and mouse were pcDNA3.1-HNF1α/GATA4/FOXA3/HNF4α (homo or mmu). The psiCHECK2-Glis2 (homo or mmu) promoter was used as the Glis2 promoter sequence vector. The following plasmid groups were cotransfected into 293 T cells, and 3–5 parallel repeat wells were set. The transcriptional activity of Glis2 by HNF1α/GATA4/FOXA3/HNF4α was detected following the kit instructions (Promega, cat. no. E1910) on a multifunctional microplate (BLT, cat. no. Lux-t020) to detect the transcriptional activity of HNF1α/GATA4/FOXA3/HNF4α for Glis2. The grouping settings were as follows: psiCHECK2 + pcDNA3.1-NC; psiCHECK2-Glis2 (homo or mmu) promoter + pcDNA3.1-NC; psiCHECK2-Glis2 (homo or mmu) promoter + pcDNA3.1-HNF1α/GATA4/FOXA3/HNF4α (homo or mmu).

Similarly, wild-type (WT) and mutant (Mut) vectors of the Glis2 promoter binding site to HNF1α were constructed: psiCHECK2-Glis2-promoter (WT or Mut). The following plasmid groups were cotransfected into 293 T cells, and 3–5 parallel repeat wells were set to detect the binding site of HNF1α to the Glis2 promoter. The specific procedure was the same as before. The grouping settings were as follows: psiCHECK2-Glis2-promoter (WT/Mut) + pcDNA3.1-NC and psiCHECK2-Glis2-promoter (WT/Mut) + pcDNA3.1-HNF1α.

### EMSA analysis

Nuclear extracts were prepared from cells using a nuclear extraction kit (Beyotime Biotechnology, P0027), and EMSA was performed using an EMSA detection kit (Thermo Fisher, #20148) according to the manufacturer’s instructions. The sequence of each probe was as follows: WT labeled: 5′-CCGGGTCCATCTGCCAGCTAATATTTAACCCGGCAGCACTGCG-3′; WT unlabeled: 5′-CCGGGTCCATCTGCCAGCTAATATTTAACCCGGCAGCAC-3′; mutated unlabeled: 5′-CCGGGTCCATCTGCCAATTGGCCAATCGGACGGCAGCACTGCG-3′.

### Statistical analysis

Statistical tests were conducted using SPSS 21.0 (SPSS, Inc., Chicago, IL, USA). Data are presented as the means ± S.D.s. For comparisons between two groups, Student’s *t* test was performed. One-way analysis of variance (ANOVA) was conducted for comparison of multiple conditions. Statistical significance was defined as *P < 0.05, **P < 0.01, ***P < 0.001.

## Results

### Glis2 is downregulated in fibrotic liver tissues and activated HSCs

**Fig. 1:**
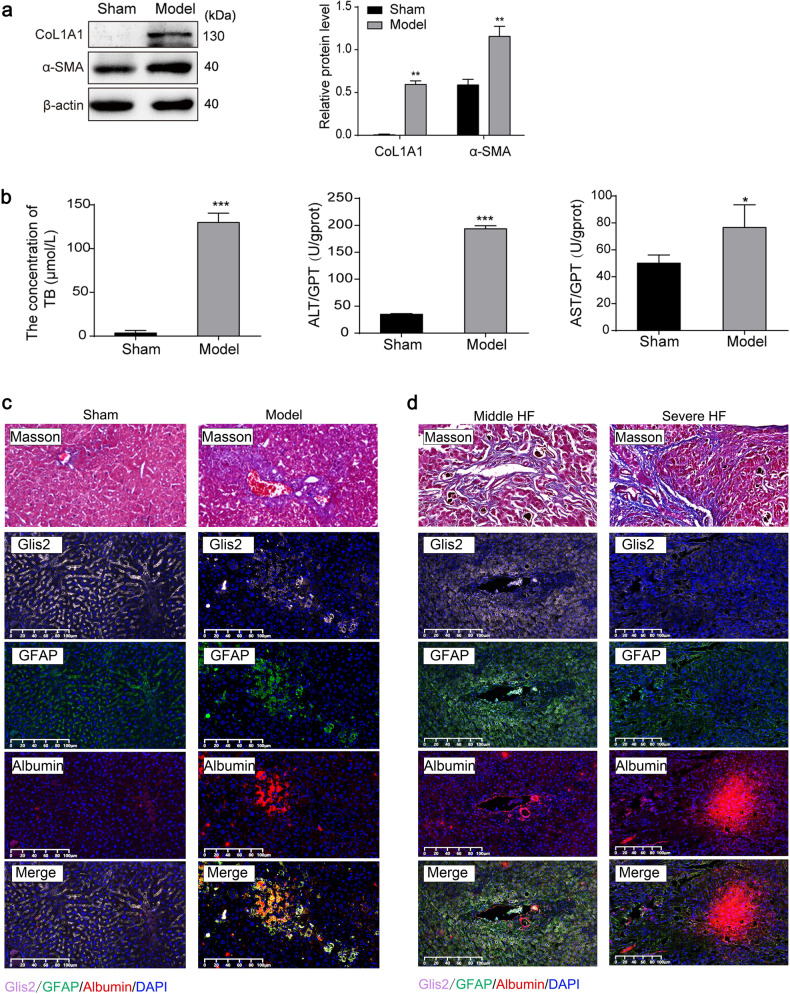
Glis2 is downregulated in fibrotic liver tissues. **a** Relative protein expression detected through western blotting. **b** Serum levels of TB, ALT, and AST detected via biochemical kits. **c** Representative pictures of multilabel immunofluorescence in mouse liver tissue with fibrosis and no fibrosis identified by Masson’s staining: Glis2 (pink), GFAP (green), albumin (red), and cell nucleus (blue). **d** Representative pictures of multilabel immunofluorescence in patient liver tissue with middle and severe fibrosis identified by Masson’s staining: Glis2 (pink), GFAP (green), albumin (red), and cell nucleus (blue). Mean ± SD; Student’s *t* test for (**a**) and (**b**); *P < 0.05; **P < 0.01; ***P < 0.001.

We first constructed liver fibrosis model mice through the BDL method. Compared with that of the sham operation group, the protein expression of collagen alpha-1 (CoL1A1) and alpha-smooth muscle actin (α-SMA) in the liver tissue of the mice in the model group was significantly increased (Fig. 1a), and the contents of liver injury biomarkers (total bilirubin (TB), ALT, and AST) in peripheral blood were significantly increased (Fig. 1b). More intuitively, Masson’s staining showed obvious collagen fiber deposition in the liver tissue (Fig. 1c, d), suggesting the occurrence of liver fibrosis in the modeling group.

Immunofluorescence (IF) staining showed that Glis2 and glial fibrillary acidic protein (GFAP), a biomarker of HSCs in the resting state, were lower in mouse liver tissues with fibrosis and in human liver tissues with severe fibrosis, while albumin, a liver injury marker specifically expressed in hepatocytes, was higher. Moreover, Glis2 was widely distributed in the cytoplasm and colocalized with GFAP (Fig. 1c, d), suggesting that Glis2 may exist in HSCs and be extensively downregulated in fibrotic liver tissues.

**Fig. 2:**
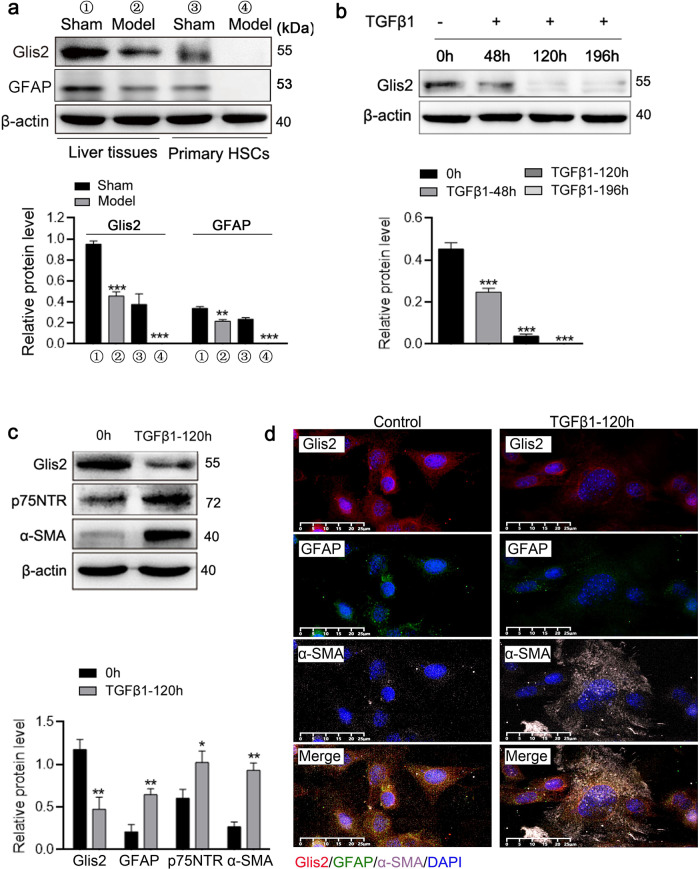
Glis2 is downregulated in activated hepatic stellate cells. **a**–**c** Relative protein expression detected by western blotting. **d** Representative pictures of multilabel immunofluorescence in JS-1 cells with or without TGFβ1 induction: Glis2 (red), GFAP (green), α-SMA (pink), and cell nucleus (blue). Mean ± SD; Student’s *t* test for (**a**) and (**c**), and ANOVA for (**b**); *P < 0.05; **P < 0.01; ***P < 0.001.

To more precisely identify the liver cell types with lost Glis2 expression, we preliminarily isolated HSCs from mouse livers. The results showed that Glis2 was downregulated in modeling samples, with greater downregulation in HSCs isolated from HF livers (Fig. 2a). Next, TGFβ1 was used to differentiate JS-1 cells, a mouse-derived hepatic stellate cell line, into myofibroblasts. Continuous stimulation with TGFβ1 (for 120 h ~ 196 h) led to a stable decrease in Glis2 protein levels (Fig. 2b), but the expression of p75 neurotrophin receptor (p75NTR) and α-SMA increased in reverse (Fig. 2c). Moreover, GFAP and Glis2 in TGFβ1-stimulated JS-1 cells were decreased simultaneously, while α-SMA was upregulated through immunofluorescence assays, indicating that Glis2 expression was decreased during myofibroblast differentiation of JS-1 (Fig. 2d).

Collectively, these data prove that Glis2 is downregulated in fibrotic livers and activated HSCs.

### Upregulation of Glis2 prevents HSC activation and HF both in vivo and in vitro

**Fig. 3:**
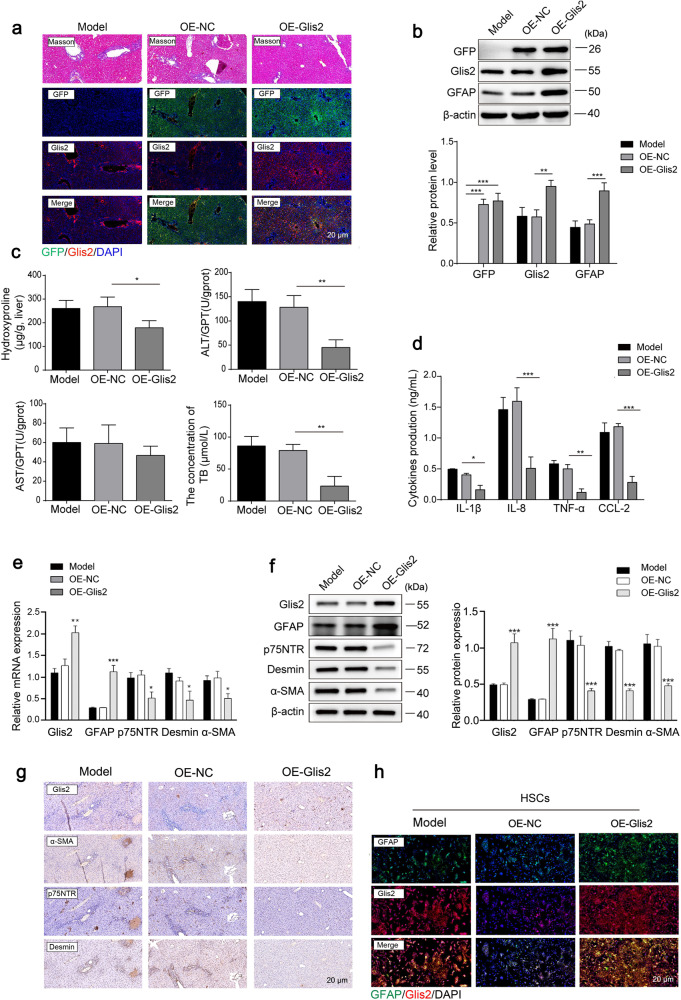
Glis2 suppresses hepatic fibrogenesis in vivo. **a** Representative pictures of immunofluorescence in mouse liver tissues with fibrosis identified by Masson’s staining: GFP (green), Glis2 (red). **b** Relative protein expression of Glis2, GFP and GFAP proteins detected in mouse liver tissues in each group through western blotting. **c** Serum levels of hydroxyproline, TB, ALT, and AST detected using biochemical kits. **d** Contents of IL-1β, IL-8, TNF-α, and CCL-2 in liver tissues detected by ELISAs. Relative mRNA and protein levels of Glis2, GFAP, p75NTR, desmin, and α-SMA in mouse liver tissues determined by qRT‒PCR (**e**) and western blotting (**f**). **g** Immunoreactivity of Glis2, α-SMA, p75NTR, and desmin in mouse liver tissues assessed by immunohistochemistry. **h** Immunofluorescence of GFAP (green) and Glis2 (red) in JS-1 cells with or without Glis2 overexpression. Mean ± SD; ANOVA for (**b**–**f**); *P < 0.05; **P < 0.01; ***P < 0.001.

BDL-induced mouse HF models were treated with recombinant adenovirus by tail vein injection to upregulate Glis2-GFP expression in vivo. Green fluorescence (GFP-derived) and upregulated GFP expression in livers were observed in stripped liver tissue (Fig. 3a, b), indicating that the adenovirus was delivered to the liver. We found that there was an improvement in liver pathology when Glis2 was imported externally, reflected in a reduction in liver injury biomarkers (ALT, AST, and TB), inflammatory biomarkers (IL-1β, IL-8, TNF-α, and CCL-2), and fibrosis biomarkers (desmin and α-SMA) (Fig. 3c–g). In addition, from the perspective of the activation degree of HSCs, the resting marker GFAP of HSCs was significantly increased after Glis2 allogenic overexpression in JS-1 cells (Fig. 3h). These findings indicate that the increase in Glis2 was conducive to the inactivation of HSCs and the reversal of liver fibrosis.

**Fig. 4:**
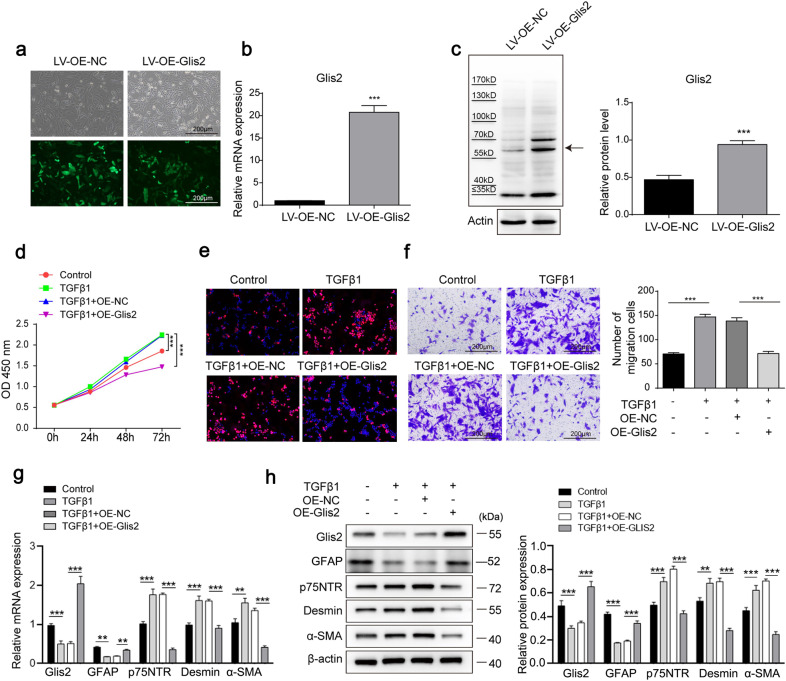
Glis2 inhibits the activation of HSCs in vitro. **a** Bright-field and fluorescence detection of Glis2-overexpressing JS-1 cells. **b**, **c** The mRNA and protein levels of Glis2 in JS-1 cells determined by qRT‒PCR and western blotting, respectively. **d** Cell proliferation monitored using the CCK-8 assay. **e** Cell proliferation was assessed using the EdU incorporation assay. **f** Cell migration detected via the Transwell migration assay. **g**, **h** Relative Glis2, GFAP, p75NTR, desmin, and α-SMA mRNA and protein levels in HSCs measured by qRT‒PCR and western blotting, respectively. Mean ± SD; Student’s *t* test for (**b**) and (**c**); ANOVA for (**d**, **f**–**h**); *P < 0.05;**P < 0.01; ***P < 0.001.

In follow-up experiments, we investigated the relationship between Glis2 expression and HSC activation in vitro. A lentivirus was used to infect JS-1 cells to establish an HSC model with Glis2 overexpression (Fig. 4a–c). TGFβ1-induced JS-1 cells exhibited a higher capacity for proliferation (Fig. 4d, e) and migration (Fig. 4f) with increased expression of activated biomarkers (p75NTR, desmin, and α-SMA) and reduced expression of the resting biomarker GFAP at both the mRNA and protein levels (Fig. 4g, h). However, these changes were significantly reversed when Glis2 was overexpressed (Fig. 4d–h), indicating that Glis2 inhibits TGFβ1-induced HSC transdifferentiation.

### Glis2 is methylated by DNMT1 in activated HSCs

**Fig. 5:**
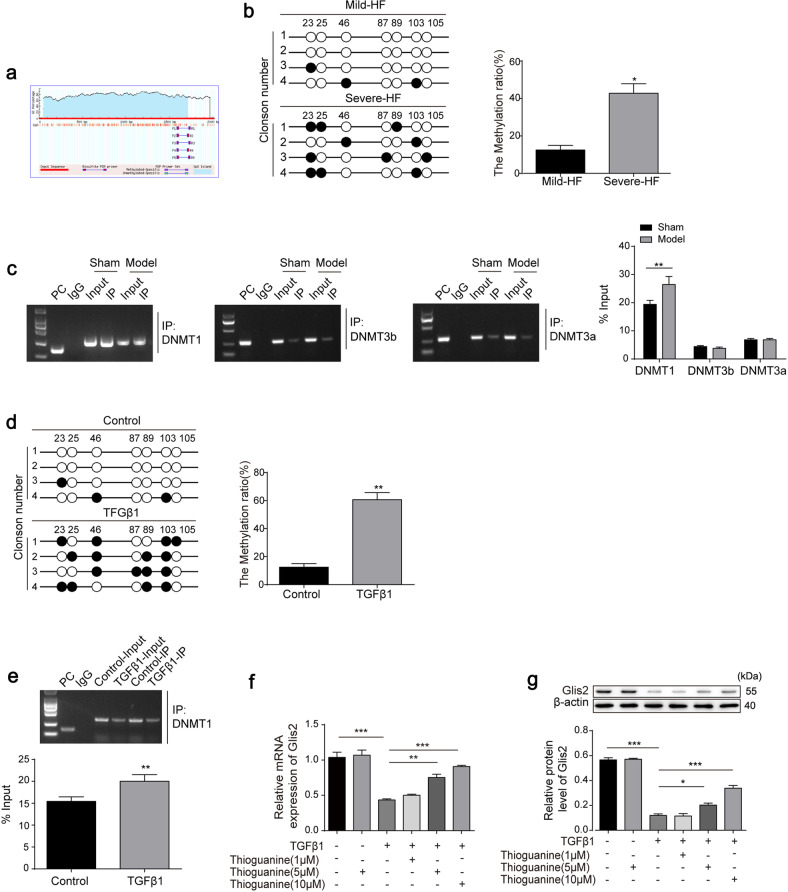
DNMT1 mediates Glis2 promoter methylation. **a** The location of the CpG island on the Glis2 promoter. **b** The methylation status of Glis2 in fibrotic liver tissues of patients detected through bisulfite sequencing. **c** The interaction between the Glis2 promoter and DNMT1, DNMT3a, and DNMT3b detected via the ChIP–qPCR assay in mouse liver tissues. **d** Methylation status of the Glis2 promoter in quiescent and activated HSCs. **e** The interaction between the Glis2 promoter and DNMT1 detected via the ChIP–qPCR assay. **f**, **g** The mRNA and protein levels of Glis2 in HSCs measured by qRT‒PCR and western blotting, respectively. Mean ± SD for (**b**–**e**); ANOVA for (**f**, **g**); *P < 0.05;**P < 0.01; ***P < 0.001.

Mounting evidence suggests that aberrant DNA methylation causes HF by suppressing the expression of key genes^12,25^. Consistently, we found an ~2000 bp long CpG island in the Glis2 promoter, 2290–4305 bp away from the transcription start site, indicating that Glis2 transcription may be regulated through DNA methylation (Fig. 5a). As expected, a higher degree of methylation was found in the Glis2 promoter in patients with more severe HF (Fig. 5b). Then, ChIP/PCR was performed to determine the abundance of the DNA methyltransferases DNMT1, DNMT3a, and DNMT3b^26^ in the Glis2 promoter in mouse liver samples. The results showed that the antibody against DNMT1 immunoprecipitated more Glis2 genomic fragments, whereas DNMT3a and DNMT3b immunoprecipitated in smaller amounts (Fig. 5c). Moreover, JS-1 cells stimulated by TGFβ1 showed a higher degree of DNA methylation in the Glis2 promoter, with more DNMT1 enrichment (Fig. 5c–e), which was gradually reversed to restore Glis2 mRNA and protein expression levels by thioguanine, a DNMT1 inhibitor, in a dose-dependent manner (Fig. 5f, g).

Taken together, these data suggest that DNMT1 is responsible for Glis2 promoter methylation, leading to a decrease in Glis2 expression in activated HSCs and fibrotic tissues.

### HNF1α loses transcriptional activity for Glis2 in activated HSCs

**Fig. 6:**
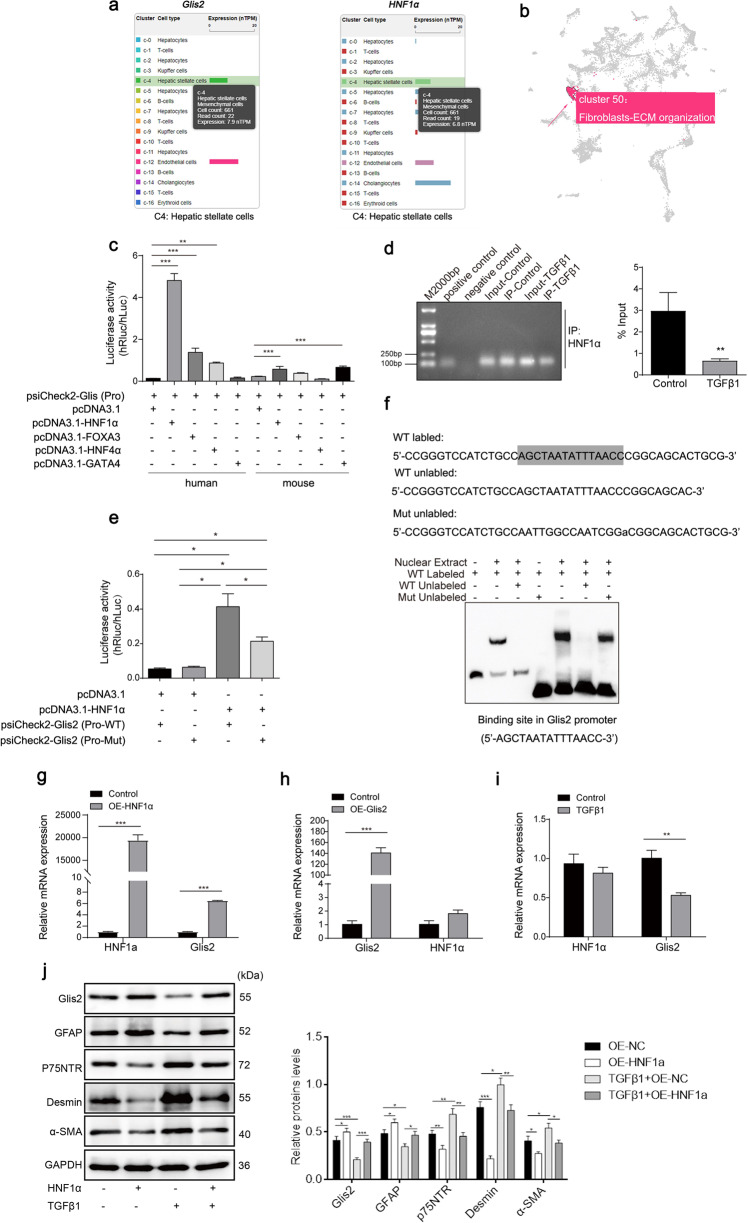
Transcriptional activation of Glis2 by HNF1α was inhibited in activated HSCs. **a** Single-cell overview of Glis2 and HNF1α in liver tissues from *The Human Protein Atlas* database (https://www.proteinatlas.org/). **b** Glis2 is part of Cluster 50 fibroblast–ECM organization with confidence by expression clustering and correlation analysis in *The Human Protein Atlas* database. **c** The transcriptional activities of Glis2 in response to different transcription factors detected by the dual-luciferase assay. **d** The binding strength in resting and active JS-1 cells verified by ChIP/PCR assay. **e** Binding motif in the Glis2 promoter region of HNF1α verified through the dual-luciferase assay. **f** Electrophoretic mobility shift assay of the binding motif. **g**, **h** Relative mRNA expression levels of HNF1a and Glis2 determined by qRT‒PCR when HNF1a or Glis2 was overexpressed in JS-1 cells. **i** Relative mRNA expression levels of HNF1a and Glis2 determined by qRT‒PCR when TGFβ1 was used to induce JS-1 for 120 h. **j** Relative protein expression levels determined by western blotting when HNF1α was overexpressed in JS-1 cells with or without TGFβ1. Mean ± SD; Student’s *t* test for (**d**, **g**, **h**, **i**), and ANOVA for (**c**, **e**, **j**); *P < 0.05; **P < 0.01; ***P < 0.001.

HNF1α is a liver-specific transcription factor. Similar to Glis2, HNF1α is mainly distributed in hepatocytes and stellate cells and is involved in the organization of the fibrous ECM (Fig. 6a, b). Here, we identified that transcriptional activation of Glis2 was significantly elevated by HNF1α and was diminished in activated HSCs. Specifically, the influence of other liver-specific transcription factors, such as GATA-4 (GATA4), liver nuclear factor 3-γ (FOXA3), and liver nuclear factor 4-α (HNF4α), on Glis2 transcription was first excluded by luciferase analysis, and the conservatism of only Glis2 activation by HNF1α was observed in both human and mouse cells (Fig. 6c). Second, HNF1α was proven to strongly bind to the Glis2 promoter (4% of input) in JS-1 cells without TGFβ1 stimulation but decreased to 1% in the stimulated cells (Fig. 6d). Next, the binding site of HNF1α on the Glis2 promoter (5′-AGCTAATATTTAACC-3′) was identified through mutation analysis through the dual-luciferase assay and electrophoretic mobility shift assay (EMSA) (Fig. 6e, f). Notably, the mRNA levels of HNF1α and Glis2 were significantly increased in the HNF1α-overexpressing JS-1 cells; however, no significant change in HNF1α mRNA levels was observed in the TGFβ1-induced Glis2-overexpressing cells (Fig. 6g–i). In addition, the upregulation of HNF1α in JS-1 cells to activate transcription of Glis2 effectively combated TGFβ1-induced myofibroblast differentiation, which was similar to the effect of direct overexpression of Glis2 (Fig. 6j). Thus, given that TGFβ1 stimulates increased DNA methylation in the Glis2 promoter, a reasonable explanation would be that hypermethylation prevents HNF1α binding to the Glis2 promoter. Thus, we can conclude that HNF1α is the upstream transcriptional activator of Glis2 in HSCs.

### MALAT1 affects HF and HSC status by recruiting DNMT1 to promote Glis2 promoter methylation

**Fig. 7:**
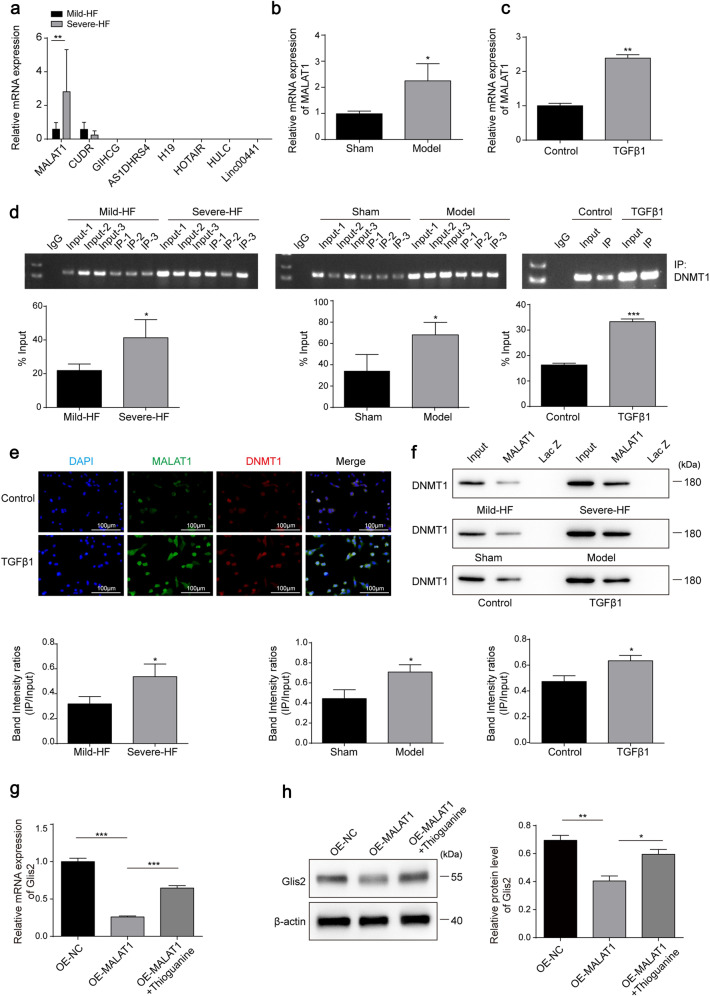
MALAT1 represses Glis2 expression by recruiting DNMT1. **a** Relative expression levels of lncRNAs in fibrotic liver tissues determined by qRT‒PCR (*n* = 6). Relative mRNA levels of MALAT1 in mouse liver tissues (**b**) and HSCs (**c**) determined by qRT‒PCR. **d** The direct interaction between MALAT1 and DNMT1 verified by RIP-qPCR in human liver tissues, mouse liver tissues and JS-1 cells. **e** Subcellular colocalization of MALAT1 and DNMT1 observed by FISH in combination with IF. Red, DNMT1; green, MALAT1; blue, DAPI. **f** The direct interaction between MALAT1 and DNMT1 determined in human liver tissues, mouse liver tissues and JS-1 cells via the RNA pulldown assay. **g**, **h** Relative mRNA and protein levels of Glis2 measured by qRT‒PCR and western blotting, respectively. Mean ± SD; Student’s *t* test for (**a**–**d**, **f**), and ANOVA for (**g**, **h**); *P < 0.05; **P < 0.01; ***P < 0.001.

DNA methylation is associated with lncRNA-mediated HSC activation in HF^13,14^. In liver disease, lncRNAs such as MALAT1^18^, linc00441^27^, HOTAIR^28^, H19^29^, CUDR^30^, AS1DHRS4^31^, GIHCG^32^, and HULC^33^ have been reported as methylation regulators. Therefore, we first evaluated the expression levels of these lncRNAs in HF tissues from patients and only detected MALAT1 and CUDR. Only MALAT1 showed significant improvement in expression in severe human HF tissues (Fig. 7a), BDL-induced mouse HF tissues (Fig. 7b) and TGFβ1-induced JS-1 cells (Fig. 7c), with remarkable elevation of binding strengths and subcellular colocalization with DNMT1 (Fig. 7d–f). Regarding the influence of Glis2 expression, upregulation of MALAT1 significantly reduced Glis2 mRNA and protein expression in JS-1 cells, while thioguanine, a DNMT1 inhibitor, blocked the effects of MALAT1 on Glis2 (Fig. 7g, h). The data in this section suggest that DNMT1-dependent Glis2 expression is regulated by MALAT1 in HSCs.

**Fig. 8:**
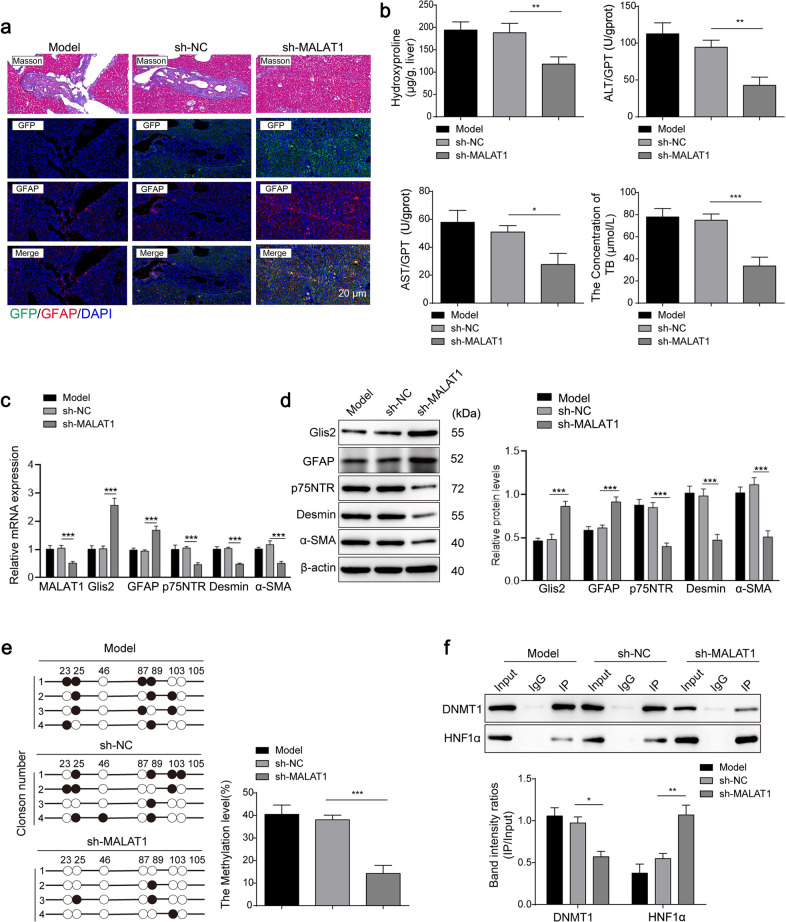
Downregulation of MALAT1 reduced Glis2 promoter methylation in vivo. **a** Pathological changes in the liver tissues of mice (*n* = 5). **b** The contents of injury biomarkers in liver tissues of mice in each group. **c** Relative mRNA expression levels of MALAT1, GFAP, Glis2, p75NTR and α-SMA. **d** Relative protein expression levels of MALAT1, GFAP, Glis2, p75NTR and α-SMA. **e** Analysis of DNA methylation sites in the Glis2 promoter through bisulfite sequencing. **f** The enrichment of HNF1α and DNMT1 in the Glis2 promoter analyzed by DNA pulldown or western blotting. Mean ± SD; ANOVA; *P < 0.05; **P < 0.01; ***P < 0.001.

In mice with liver fibrosis induced by BDL, adenovirus with MALAT1 interfering RNA was injected through the tail vein to evaluate the effects of MALAT1 on HF. Remission of HF symptoms, including histopathological improvements (Fig. 8a) and reduced levels of biomarkers of liver injury and fibrosis, was observed in the livers of mice with MALAT1 interference (Fig. 8b–d). For Glis2, its mRNA and protein levels were increased significantly (Fig. 8c, d), and the methylation modification in the promoter region was decreased with less DNMT1 but more HNF1α binding (Fig. 8e, f).

**Fig. 9:**
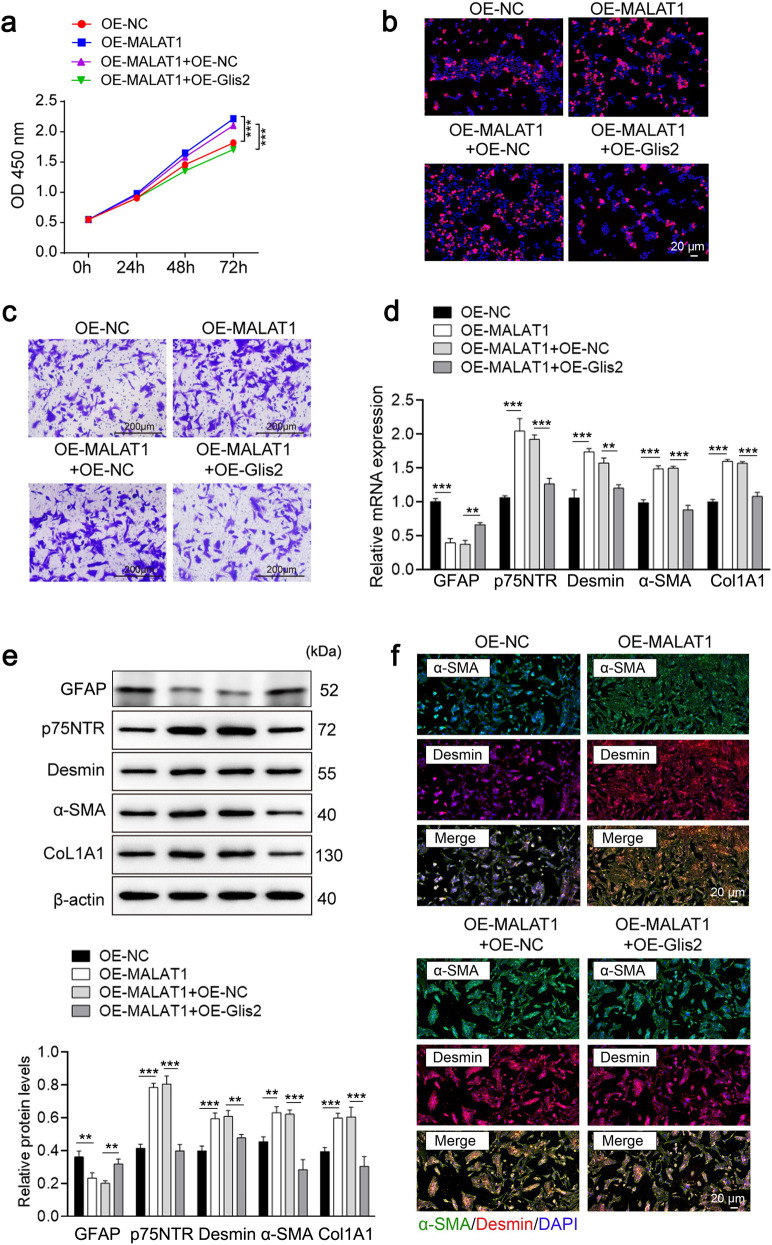
MALAT1 promotes hepatic fibrogenesis in vitro by regulating Glis2. **a** Cell proliferation monitored via the CCK-8 assay. **b** Cell proliferation assessed using the EdU incorporation assay. **c** Cell migration detected using the Transwell migration assay. **d**, **e** Relative mRNA and protein expression levels of GFAP, p75NTR, desmin, α-SMA and CoL1A1 in HSCs determined by qRT‒PCR and western blotting, respectively. **f** IF staining of desmin and α-SMA in HSCs. Mean ± SD; ANOVA; **P < 0.01; ***P < 0.001).

To test the role of the MALAT1/Glis2 regulatory axis in HSCs, we overexpressed MALAT1 either alone or in combination with Glis2 in TGFβ1-induced JS-1 cells. Upregulation of MALAT1 significantly restored the proliferative ability (Fig. 9a, b), migratory capacity (Fig. 9c), and expression of activated HSC markers such as p75NTR, desmin, α-SMA, and CoL1A1 in JS-1 cells, and these factors were significantly repressed when Glis2 was overexpressed (Fig. 9d–f), indicating that the MALAT1/Glis2 regulatory axis significantly affects the status of HSCs cultured in vitro.

**Fig. 10:**
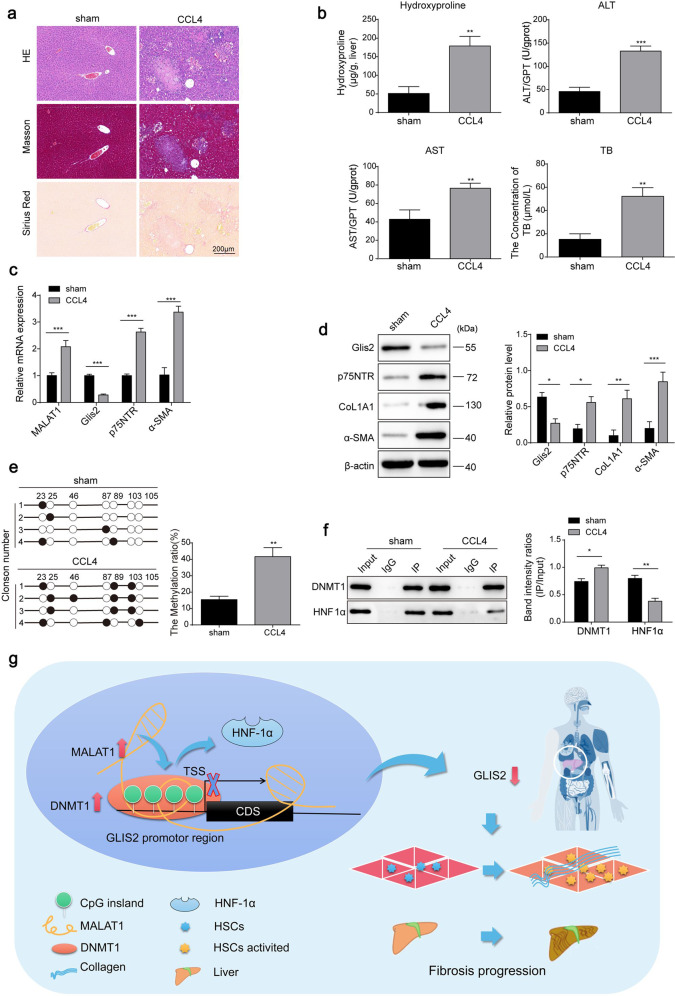
The MALAT1/Glis2 regulatory axis was established in the CCl4-induced mouse hepatic fibrosis model. **a** Pathological changes in liver tissue in mice (*n* = 5). **b** Injury biomarkers in the liver tissues of mice in each group. **c**, **d** Relative mRNA and protein expression levels measured by qRT‒PCR and western blotting, respectively. **e** Analysis of DNA methylation sites in the Glis2 promoter through bisulfite sequencing. **f** The enrichment of HNF1α and DNMT1 in the Glis2 promoter analyzed by DNA pulldown or western blotting. Mean ± SD; Student’s *t* test; *P < 0.05; **P < 0.01; ***P < 0.001. **g** Graphical abstract of the transcriptional regulation of Glis2 in hepatic fibrogenesis.

Next, to investigate whether the regulatory mechanism involving the MALAT1/Glis2 axis in HF is model specific, we developed the commonly used CCl4-induced HF model in mice. Fibrotic lesions were evaluated through tissue section staining, including H&E, Masson’s, and Sirius Red staining (Fig. 10a), as well as by examining liver injury and fibrosis biomarkers (Fig. 10b). Contrary to the results from biomarker analysis of fibrosis and MALAT1 evaluation, Glis2 mRNA and protein expression was significantly downregulated in fibrotic liver tissues (Fig. 10c, d). Moreover, we observed a significant increase in Glis2 promoter methylation, accompanied by an increase in DNMT1 binding and a decrease in HNF1α binding (Fig. 10e, f). This finding is consistent with the results obtained from BDL-induced HF, implying that different HF modeling methods share a MALAT1-mediated mechanism involving inhibition of Glis2 expression.

Collectively, these data demonstrate that MALAT1 is upregulated in the fibrotic liver and induces DNA methylation by recruiting more DNMT1 to the Glis2 promoter, thereby transcriptionally repressing Glis2 expression and activating HSCs to aggravate HF (Fig. 10g).

## Discussion

HF involves an imbalance in the synthesis and degradation of ECM in liver tissue. Various chronic pathogenic factors cause excessive deposition of ECM, leading to abnormal liver structure and function^34^. HF is a common step in the progression of chronic liver disease to cirrhosis or even liver cancer^35^. However, the etiology of HF is complex, and it is challenging to obtain sufficient clinical samples for study. Therefore, much remains unknown about the molecular mechanism of HF development.

In this study, pathological tissue samples from patients with HF were first tested to identify lower Glis2 expression in fibrotic tissues, and this finding was then verified in the mouse model of HF and TGFβ1-induced HSCs. Subsequently, we identified the involvement of MALAT1 in the DNA methylation of the Glis2 promoter through the recruitment of DNMT1. Moreover, the liver-specific transcription factor HNF1α was found to be responsible for maintaining the high expression level of Glis2 in resting HSCs, which failed to activate when the Glis2 promoter was hypermethylated. In summary, we demonstrate that the HF process is accompanied by the loss of Glis2 expression and that upregulated Glis2 contributes to the remission of the disease. Mechanistically, MALAT1 promotes Glis2 methylation by recruiting DNMT1 and blocks the transcriptional activation of Glis2 by HNF1α, thereby lowering Glis2 expression in HSCs to activate myofibroblast transdifferentiation.

Many lncRNAs have been reported to play important roles in liver diseases, but only MALAT1 and CUDR were detected to be expressed in fibrotic liver tissues of clinical patients, and only lncRNA MALTA1 was overexpressed in samples with severe fibrosis, suggesting that the expression of lncRNAs in different types of liver diseases is quite different. However, there are limitations in the number of samples of patients with HF, and our results can be used as a reference. Furthermore, we found by dual luciferase assays that among the liver-specific transcription factors HNF1α, HNF4α, FOXA3, and GATA4, only HNF1α‘s transcriptional activation of Glis2 was conserved between human and mouse species (indicating the need for a large number of experiments in mice). Unlike some transcription factors that bind by recognizing methylation sites and then mediating demethylation to promote transcription, HNF1α‘s recognition of Glis2 promoter binding sites is limited by the degree of Glis2 promoter methylation, a common mode in which promoter methylation blocks the action of transcription factors^36^. In addition, overexpression of HNF1α can partially suppress TGFβ1-induced myofibroblast transdifferentiation of JS-1 cells, which is similar to the upregulation of Glis2. In conclusion, HNF1α is the transcriptional activator of Glis2 in the liver, and its effect is decreased when the promoter region is methylated.

Some studies have shown that Glis2 has a significant inhibitory effect on renal fibrosis^9,10,37^; however, as mentioned above, the role of Glis2 in HF is controversial. Loft et al. suggested that Glis2 knockout mice were less likely to develop liver fibrosis after diet induction with a high-fat and high-sugar diet^11^, which is partly contradictory to our findings. We believe that this discrepancy may because the biological function of Glis2 that has not been fully elaborated and the different model induction methods. Glis2 functions primarily as a suppressor of transcription^38^ and suppresses cell reprogramming^39^. Therefore, Glis2 may be necessary to maintain the differentiation stage in cells. In the present study, we found that Glis2 can help prevent the accumulation of collagen fibers in the liver after chronic injury by inhibiting the myofibroblast transdifferentiation of HSCs. However, these findings were also limited to stellate cells, and liver tissue is one of the most complex organs in the body; therefore, more work is needed to fully assess the global effects of Glis2 on HF.

Collectively, this study provides a regulatory mechanism for Glis2 expression in HSCs from the perspective of DNA methylation mediated by lncRNA MALAT1. Reducing the expression of MALAT1 or removing the promoter methylation modification of Glis2 to ensure its expression level in HSCs is beneficial to alleviate HF.

## Data Availability

All data generated or analyzed during this study are included in this article. The datasets used and/or analyzed during the current study are available from the corresponding author on reasonable request.
