# Author Correction: Transcriptional regulation of Glis2 in hepatic fibrosis

**DOI:** 10.1038/s12276-023-01061-6

**Published:** 2023-07-21

**Authors:** Huan-Yu Gong, Peng-Cheng Zhou, Hao-Ye Zhang, Li-Min Chen, Yang-Mei Zhou, Zhen-Guo Liu

**Affiliations:** 1 Department of Infectious Disease, the Third Xiangya Hospital, Central South University, Changsha, 410013 Hunan PR China; 2 Hunan Key Laboratory of Viral Hepatitis, Xiangya Hospital, Central South University, Changsha, 410008 Hunan PR China

**Keywords:** Cell biology, Cancer

Correction to: *Experimental & Molecular Medicine* 10.1038/s12276-023-01031-y, published online 03 July 2023

The Funding information section was missing from this article and should have read ‘This study was funded by grants from the National Natural Science Foundation of China (No. 82170639), and the Hunan Provincial Natural Science Foundation (No. 2022JJ70068, 2022JJ70070)’.

The original article has been corrected.

